# Yellow Fever, Considered in Its Historical, Pathological, Etiological, and Therapeutical Relations, Including a Sketch of the Disease as It Has Occurred in Philadelphia

**Published:** 1855-11

**Authors:** 


Art. II.—*Yellow Fever, considered in its Historical, Pathological, Etiological, and Therapeutical Relations, including a Sketch of the Disease as it has occurred in Philadelphia, from* 1699 *to* 1854, *with an examination of the connections between it and the fevers known under the same name in other parts of Temperate as well as in Tropical Regions.* By R. La Roche, M. D., Member of the American Philosophical Society; of the American Medical Association; Fellow of the College of Physicians of Philadelphia; Corresponding Member of the Imperial Academy of Medicine, and Foreign Associate of the Medical Society of Emulation, of Paris; of the Academies of Sciences of Turin, Copenhagen, Stockholm, Nancy, and New Orleans, of the Medical Societies of Naples, Marseilles, Lyons, etc. In 2 vols. Philadelphia: Blanchard & Lea. 1855.

A question in regard to yellow fever which, just at this time, is exciting special concern, is the extent to which the epidemic is likely to spread in a northern direction. It has prevailed as far north as Memphis during the present season; will it spread farther in future years? Hitherto it has been regarded as a disease of low, flat localities in hot latitudes, but it has on many occasions reached far into the temperate zone. Dr. La Roche says:

"Until recently, the river Amazon, which divides Brazil from Guiana, formed the boundary of the disease south of the equatorial line; for, although it is said to have prevailed at Olinda from 1687 to 1694,* and to have shown itself as far as Montevideo† in the beginning of the present century, the latter circumstance is open to some doubt; while, in Brazil, from the close of the 17th century to the middle of the present, the disease was not observed. Since 1850, it has invaded Rio Janeiro, Bahia, Pernambuco, and other places of that country. It is, in greater measure, a stran-

* Ferreyra da Rosa, op. cit. † Humboldt, Nouvelle Espagne, p. 761.

ger to the Pacific, having prevailed but once at Panama,* twice at Guayaquil, and once at Callao. It does not appear in the East Indies; the fever described by Wade, Fontane Lind (of Windsor), Johnson, Twining and other writers appertaining to the class of common bilious remittents. It has never prevailed in China, Cochin-China, Singapore, Siam, Ceylon; it has prevailed only occasionally on the African coast, Senegal, and the Gold Coast, and has but three times, in the space of eighty-six years, showed itself in Cayenne.

"Within those limits, it has in some one or more places originated and prevailed to a greater or less extent—occasionally or frequently—either as an endemic or as a mild or wide-spreading epidemic.† Beyond these it never shows itself; and though—whether north or south, east or west—it does not reach the point at which common malarial fevers stop, it approximates to these diseases, so far, especially, as its northern or western extension is concerned, in being circumscribed within certain bounds; for they, too, have their limits. The effect in both instances is due to modifications in the same morbific agencies."‡

Dr. E. H. Barton, who has been a careful observer of the progress of yellow fever, states that within the limits of the zone above pointed out, the disease usually commences its epidemic career in the South, and advances gradually towards the North. Starting in the West Indies, South America, or Mexico, one year with another, about the first of May, it is two months reaching New Orleans, and as a general rule does not break out as an epidemic in our middle States or in Europe before the latter part of July or the first of August. Temperature seems to be the controlling cause, but it must be admitted that the etiology of the disease is still obscure. The mean summer heat of Philadelphia and of New York is not less in latter years than it was at the close of the last century when the fever was so rife in those cities. The towns on the Mississippi have the same climate now that existed when they were founded, and yet yellow fever, not dreaded then, has within a few years become an annual scourge of those communities. Climate alone will not account for the change. Heat and moisture, and even favoring vegetable decomposition hardly afford a satisfactory explanation. The pestilence is enlarging its dominions, but what it is that has imparted to it this

* Humboldt, loc. cit.

† De la Condamine, Voyage à l'Equateur, Paris, 1751, vol. iv. Ulloa, Voyage Historique de l'Am. Mérid., i. p. 149, 1752. Jameson, Rept. of San. Con. of New Orleans, 1853, p. 141.

‡ Leblond, Obs. sur la Fièvre jaune, p. 200

unwonted activity, has not been explained. Will this augmented activity continue, and will the fever extend its march towards the North, and invade places hitherto secure from its incursions? Time only can return an answer to this question, but the possibility of such an occurrence imparts an interest to yellow fever which the subject never before possessed with the profession.

Dr. La Roche gives a minute account of the symptoms of yellow fever, in reading which one is particularly struck with the protean character of the disease. Unlike cholera, the features of which are so uniform, few cases of yellow fever exactly resemble each other. It is impossible to present a satisfactory view of the symptoms in the limits at our disposal, and so we pass the subject over entirely, referring the reader to the work for full information.

Yellow fever, Dr. La Roche continues, is everywhere the same disease, in tropical and in temperate climates, and in this opinion he is sustained by the general testimony of those who have written on the subject. There are those, however, who insist that the fever in tropical regions is a disease entirely different from that which has been designated yellow fever in temperate latitudes. It would seem that this is a question not easily decided, seeing that the fever is one of so variable a character, and yet we have no doubt that it is essentially the same disease wherever it appears. It may not be practicable in a few words to describe its pathognomonic symptoms, but that it has features by which those who have seen it once never fail to recognize it afterwards, is the testimony of nearly all who have made observations on the subject. Dr. La Roche devotes several pages to the proof of this position, and by comparing the tableaux of the phenomena presented by the fever in the various regions where it has prevailed, he has rendered the point exceedingly clear.

He next proceeds to examine in detail the phenomena presented by the several organs of the body, and first those of the circulatory system. The blood has attracted much attention in yellow fever. Dr. Rush noticed in the epidemic of 1793, that the usual separation into serum and crassamentum did not take place, and that in other cases where serum was separated, it was of a yellow color; while in others, again, the blood was as sizy as in pneumonia or rheumatism, and still in others, was of a dark color, and as fluid as molasses. In some patients, he adds, "the blood in the course of the disease, exhibited nearly all the appearances which have been mentioned. They varied according to the time in which the blood was drawn, and the nature and force of the remedies which had been used." Dr. Physick "found the blood in the heart in a fluid state, and similar to that of persons who have been hung or struck by lightning." Numerous observers testify to the fluid condition and discolored appearance of the blood, when issuing from the natural outlets of the system in the closing stages of the disease. In the epidemic of 1805, in Philadelphia, Dr. Caldwell remarked that the blood which flowed in hemorrhage was never capable of firm coagulation, thereby evidencing "that its vitality was nearly extinguished," and that in many cases it was dissolved.

"A diseased condition of the blood, similar to the one noticed in the yellow fever of this city, had long before been recorded, with more or less minuteness, by writers in this and other countries. Lining states that, in the fever of Charleston, 'the blood saved at venesection had not any inflammatory crust. In warm weather it was florid, like arterial blood, and continued in one soft homogeneous mass, without any separation of the serum after it was cold. When there was any separation, the crassamentum was of too lax a texture.'* In a subsequent page, he speaks of a partial dissolution of the blood in the after-stages of the disease, as exhibited in the fluid discharged in various hemorrhages.† Moultrie states that, in the first stage, the blood taken in venesection is dissolved, the crassamentum is broad, thin (*crassamentum ejus latum minimeque profundum*), floating on the surface. It is of less firmness than in the natural state, and exhibits, at times, livid spots on the surface. In the last stage it is black, dissolved, and in a state of putrefaction (p. 7). From that day to this, all writers on the fevers of this country, with scarcely one exception, mention incidentally or call special attention to the altered state of the blood throughout the whole course of the disease, or during its latter stages; its defective coagulability, the absence of inflammatory crust; the softness of the coagulum; the absence of separation; the peculiar appearance or small proportion of the serum; the dark color of the whole mass; its altered consistence, etc."‡

* Edinb. Med. Essays and Obs., ii. 415. † Ibid., p. 422.

‡ S. Brown, p. 15; Valentin, p. 182; Irvine, p. 25; Miller, p. 62; Mitchell, Med. Mus., i. 6, 7; Kelly, Am. Jour. (N. S.), xiv. 389; Harrison, N. O. J., ii. 139; Stone (F. of Natchez), N. O. J., vi. 571; Ib., N. O. J., ii. 181; Cartwright, Med. Rec., ix. 26–7; Drysdale, Med. Mus., i. 138; Gros, F. of N. O. in 1817, p. 11; Dalmas, p. 16; E. H. Smith, p. 122; Archer, Med. Rec., v. 68; Nott, Am. J., N. S., ix. 281; Townsend, p. 196; Cooke, N. O. J., x. 621; Lewis, ib., i. 301.

Some of the conclusions at which Dr. La Roche arrives on a survey of all that has been written on this subject are:—that in some instances the blood, during the first stage of the fever is unaltered, in color, the firmness of its crassamentum, or its power of coagulation; that more generally its coagulability is lost, or greatly impaired; that in some cases it is found sizy, and even cupped; that more frequently it is neither sizy nor cupped; that it is sometimes of a florid red color, at the outset of the disease, but always grows dark as the malady advances; that in some instances it has been found, even at the outset, quite fluid and of thin consistence, while in others it is represented to have been thicker than in health.

"But these are not the only changes that are found to occur in the blood of yellow fever. M. Chassaniol, whose residence, during several years, at Guadaloupe, afforded him ample opportunities of investigating the subject, has recently communicated the result of sundry experiments on the blood, instituted at his request by an able chemist, and which led to the discovery that this fluid after death, and most probably in the latter stages of the disease, contains a notable quantity of urea. In one case, 50 gram. of blood, taken from the heart a few hours after death, gave 0·24 of that substance, and, in another, 60 grs. gave 0·29. In stating these results, the operator remarks that the blood examined must have contained a larger quantity of urea than here mentioned, as a portion, doubtless, escaped with the albumen at the moment of coagulation. M. Chassaniol is of opinion that the phenomena of the second stage are due to the poisoning of the blood by this substance.* It may not be improper to remark that urea has been detected in the blood of the relapsing fever of Scotland, as also in the serum contained in the ventricles of the brain;† that, in this disease, as in the yellow fever, the urinary secretion is impaired, and that in the former many of the symptoms of the last stage have been satisfactorily traced to the poisoning action of that substance on the blood, and of the latter on the brain and other important organs. Nor should we lose sight of the fact, that urea usually exists in increased quantities in cholera—the amount differing considerably in the several stages of the disease; that it is small in quantity in the intense stage of collapse; increases during reaction, and is in excess when consecutive febrile symptoms occur.‡ In this disease, as in yellow and relapsing fever, the urinary secretion is greatly impeded, and we naturally

* Compte rendu de l'Ac. des Sci. Meeting of 12th Dec., 1855.
† Henderson, Edinburg Journ., lxxii. 223, etc.
‡ Reports on Epid. Cholera, Roy. Coll. of Phys., 2d Rep. by D. Gull, pp. 51–3.

infer that, in all, the presence of urea in the blood is due, in part at least, to want of elimination of it through means of its natural emunctory, and, to some degree, to a disordered condition of the secretory function."

Under the head of "circulatory system," our author treats at length of the pulse and hemorrhages, and then proceeds to describe the state of the skin, as regards temperature, color, sensibility, secretion, odor, and diseases. Next the "digestive organs" are considered, and in connection with them that remarkable phenomenon of yellow fever, black vomit, which by many writers has been held to be the most distinctive, the pathognomonic symptom of the disease. From the prominence of this feature it is that the Spaniards have denominated yellow fever *vomito prieto.* Nevertheless, black vomit is not peculiar to this fever, nor is it of itself enough to stamp the case in which it occurs as one of yellow fever. Black vomit may result from the action of various poisons, or from the introduction of putrid substances into the cirlulation, and is occasionally an attendant upon puerperal and other fevers. Dr. Dickson says he has met with it in bilious remittent fever; Cleghorn saw it in the tertian fever of Minorca; and Andral remarked it in some cases of fever which occurred, in 1822, in the hospitals of Paris. It has been met with in cases of typhus, in oriental plague, in *coup de soleil*, in colic, and in a case of psoas abscess. The writer of this article attended a case of Bright's disease in a child, a few weeks since, in which there was vomiting of a black matter several days before the death of the patient.

These examples will suffice to show that black vomit is an occasional occurrence in many affections differing widely from yellow fever, and therefore not of itself sufficient to characterize that disease. And yet it is far more frequently an attendant upon this fever than upon any other complaint. It usually makes its appearance at the opening or about the middle of the second stage of the disease, sometimes at the decline of the first stage, more rarely during the first paroxysm; in other words, about the fourth or fifth day. It is a highly unfavorable sign; more so than the jaundiced condition of the skin, but yet not always a fatal symptom, though, according to Dr. Fenner, not more than one in a hundred recovers where it is distinctly pronounced.

"The black vomit, notwithstanding its name, is rarely of a black color. As seen in this city, it is more frequently of a dark-brown, bister, chocolate, or umber hue. In some instances, the color approaches to a dark slate, or to a muddy claret. It is of two kinds. The one consists of a number of dark, flaky particles, which have been not unaptly compared to butterfly or bees' wings, and which assume gradually the appearance, with more or less distinctness, of the grounds of coffee, of soot, or finely-powdered charcoal, floating in a quantity more or less considerable, of thin, glairy fluid bearing a slight resemblance to a weak infusion of flaxseed or green tea. The latter fluid, when filtered, differs slightly in color, being limpid like water, of a deep brandy or rum color, yellowish or light green. The afore-mentioned flakes or striæ are at first, or throughout the milder forms of the disease, limited in point of number, and of a light or grayish slate or chocolate tinge. But as the disease advances, and especially in the more malignant cases, they increase in number, and become darker and darker till the whole appears uniformly blackish or even black. The fluid, when completely formed, though homogeneous in appearance when discharged, separates soon, on standing, into two parts; the one consisting of the flaky or coffee-ground matter already mentioned, and the other of the fluid in which they were held in suspension. This flaky matter, which in some cases seems collected in masses of greater extent and entangled in mucus, and at others is divided very minutely and equally mixed, after subsiding to the bottom of the vessel, either in distinct particles or in the form of a dark powder, is readily incorporated with the fluid by the least agitation. When the black vomit is kept a long while, its constituents become perfectly separated, but when shaken and reincorporated, they show less disposition to separate again. In some instances, the quantity of the fluid portion is, from the outset to the close of the attack, very large compared to the solid particles. In others, the reverse is the case; the substance vomited, though presenting all the characteristic features of the coffee-ground or granular matter, being almost or completely deprived of its fluid attendant. In a case I had occasion to see in July, 1853, with Dr. Keating, the black vomit presented that character. In another case, the proportion of solid matter in the fluid thrown up was very small; while, on dissection, sixteen hours after death, the stomach was found to contain about six ounces of thick coffee-ground or granular matter, with scarcely any admixture of fluid.

"The other form of the black vomit is more homogeneous in character, and presents the appearance of dark-colored inspissated mucus or thin tar, or of a thick mixture of molasses and water. In some instances, the matter vomited consists of grumous or dark-colored blood, fluid or coagulated, without admixture of coffee-ground particles or pale fluid;* while in others, again, the matter described above is mixed with coagula of more or less pure blood; or, failing to maintain the character above described, assumes more or less the appearance of blood. In some instances, the discharge towards the termination of the disease becomes nearly sanguineous."

What is the source and nature of black vomit? This question has excited much prolix discussion, but we pass over it all to give the views of our author respecting it, which will be gathered from the following extract:—

"Enough has now been said to prove beyond doubt that the black vomit is neither altered bile nor the result of a secretory action on the part of the gastro-intestinal capillary vessels. In view of this, and taking into consideration various facts to which attention is next to be called, we shall be led to the conclusion that it is nothing but blood in a peculiar state of alteration. That it is the product of the stomach itself, when ejected from that organ, or of other surfaces whence it may proceed—the intestinal, urinal, pleural, peritoneal, etc., has been shown. That the black vomit, or a fluid like it, proceeds from the capillary vessels of these parts; that it may be, and has been pressed out of those vessels after death, and is often seen then and during life oozing out of the capillaries of parts accessible to sight, as the fauces, nostrils, skin, and eyes, are facts which, as we have seen, are too well established to be denied, and must of themselves go far to prove the sanguinous nature of the fluid, especially when we bear in mind the reasons assigned for rejecting the idea of its being the product of a secretory action of those vessels. For, if we refuse to admit that the fluid issuing from or pressed out of these has been secreted by them, we have no alternative but to regard it as blood; the only substance contained in those vessels, whether in health or disease. To the same conclusion we naturally arrive when we take into consideration the fact that the black vomit is the product, in some cases, of gastric inflammation occasioned by the ingestion of acrid poisons, or by other causes; and we know that in inflammation resulting from such agencies—or indeed from any agency—the vessels are engorged with blood, and nothing else; and that, therefore, what proceeds from them, whatever peculiar appearance it may assume, must be blood also. The same must be true as regards the contents of the capillary vessels in inflammation or congestion of the stomach or other parts in yellow fever. If we find these filled with red or dark fluid, and

---

* Cathrall, Analysis of Black Vomit, pp. 4, 6, 7; Rush, Yellow Fever of 1793, p. 61; Wood's Practice, i. 301; Monges, N. A. Med. and Surg. Jour., ii. 57.

if their engorgement and dark color are relieved by the discharge of that fluid, we may safely infer that the latter was blood, for hemorrhage is one of the common modes in which inflammation and congestion have a tendency to terminate; and if the red or daık fluid which occasions the relief in ordinary cases is admitted to be of the nature in question, there can be no reason to doubt that when the effect is obtained in yellow fever, the fluid discharged is of a similar kind. Nor is this all! We have seen that black vomit very generally adheres to those portions of the mucous membrane of the stomach or intestines that are inflamed or congested, or from which it was effused, leaving other parts uncovered. In this we have a striking proof of its analogy to real blood, which exhibits a like disposition to adhere to inflamed surfaces from which it had exuded."

That this is the nature of black vomit is still more satisfactorily proved by the fact that the blood, by the addition of muriatic acid, may be made to assume the characteristic appearance of this fluid. And, to establish the position beyond the possibility of doubt, the microscope has revealed in black vomit the presence of unchanged blood-globules, and of a mass of matter resulting from their disintegration. "In a word, the microscope enables us to perceive that the sedimentary portion of the black vomit is composed almost entirely of blood—corpuscles in various stages of degradation."

We must pass over several chapters treating of the tongue, pain, respiration, countenance, urine, and state of nervous system in yellow fever, to give a summary of the anatomical characters that pertain to the disease. The post mortem appearances are:—discolored surface, stomach containing matter similar to that vomited during the latter stage of the disease, the mucous membrane generally diseased; intestines, for the most part, similarly affected; liver abnormal, yellow, brown, red, purple, bluish, slate, chocolate, or livid, but not always; spleen generally darker than usual, enlarged, and friable; kidneys generally diseased; brain, spinal cord, lungs, for the most part, normal. Do any of these appearances belong in a special manner to yellow fever? Louis thought he had succeeded in establishing that the peculiar condition of the liver is so to be regarded, but the testimony against him is irrefragable. What, then, are the anatomical characters of yellow fever? Dr. La Roche would name the following as best entitled to that rank:—a dark mahogany color of the skin; petechial condition of the gastric mucous membrane; black matter in the cavity of the stomach and bowels; a yellow discoloration and fatty degeneration of the liver; an albuminous state of the urine.

We cannot stop to make any abstract of the chapters on critical days and critical efforts, type of yellow fever, complications, duration, prognosis, and incubation, which are as full and satisfactory as any others in the elaborate work, but pass on to notice some of the last topics discussed in the first volume. One of these is the mortality of yellow fever. Dr. La Roche reviews the several epidemics which have desolated Philadelphia, and thus remarks:—

"If now we sum up the facts embraced in the foregoing statements, we shall find that exclusive of the deaths that occurred from sporadic cases in 1796, 1806–7–8, and other years, and laying aside those seasons relative to which we have no precise information, the fever has caused a loss in Philadelphia, from 1699 to the present day, during eleven visitations, of not less than twelve thousand seven hundred and fifty-one individuals, being on the average of eleven hundred and fifty-five each sickly year. In the four epidemic seasons of 1793–7–8–9, it caused a loss of ten thousand and thirty-eight. When we inquire into the ratio of mortality to the population, basing our calculations only on the information we possess relative to a limited number of years, we find it to have been 1 in 137.43 of the entire inhabitants, and 1 in 128.94 of those who remained exposed to the infection. The three epidemics of 1793, 1797, and 1798, alone give an average of 1 death in 14.24 of the entire population, and 1 in 10.03 of those that remained. The mortality among those affected, estimating it from the results recorded in each epidemic year, from 1793 downwards, varied from 1 in 1.2 (1819) to 1 in 3.86 (1805), giving an average for all those epidemics of 1 in 2.12. The loss at the hospital alone, during the epidemics of 1793, 1797, 1798, 1799, and 1803, the only years in which we find the admissions recorded, varied from 1 in 1.68 (1799) to 1 in 2 (1803), with an average for the six seasons of 1 in 1.867.

"From these results, the conclusion is inevitable that the yellow fever, as often as it has appeared among us, has occasioned as large a mortality, in proportion to the whole population, or to that portion of it on which it more particularly exercised its action, as it has been found to produce in any part of the United States or elsewhere. Few cities beyond the tropics—New Orleans, Charleston, and one or two others excepted—have been as repeatedly visited by the fever as Philadelphia was prior to 1820. In few has it spread more extensively than it did during some of our epidemics; and certainly in none, within the limits of this country, has it assumed a more malignant garb, and given rise to a greater ratio of mortality among those attacked. Nay, it may be doubted whether the records of West Indian or European epidemics exhibit more than a few instances in which the disease has proved more extensively destructive to human life. But it is to be borne in mind that the number of cases reported or estimated at each return of the disease in all probability falls far short of the true one. In all seasons, many—very many, indeed—of the mild ones are not taken heed of by their medical attendants, the health officers, or the public at large. They recover, and nothing is said about them. I know full well that such was the case in 1820 and in 1853, and there is no doubt that similar occurrences took place in former epidemics. Had all these cases been reported, and had our calculation of the mortality been based on the full amount of the individuals affected, the result would have been less painful. But, after making every allowance possible for the number of such omissions, it is not to be concealed that the proportionate mortality has ever been considerable. Painful, therefore, as the avowal may be, every candid Philadelphian must admit that much as some writers among us have glorified themselves for their extraordinary success in the management of the disease, the facts that have been laid before the reader justify the conclusion that neither during our earlier nor our late epidemics, has anything been achieved, calculated to justify a claim on our part to superiority of success."

This mortality Dr. La Roche is by no means disposed to refer to the unskillfulness of the physicians who encountered the fever at its various returns; he ascribes it to the deadly malignity of the poison which gave rise to the disease. As in cholera, and in all the fearful epidemics which have ravaged the earth, there are many cases of yellow fever "in which the patient may be properly said to begin to die from the very outset of his attack." During some of the epidemics these are the exceptional cases, but again, at its return, such cases predominate. Some physicians encountering yellow fever in its milder form have met with great success in its treatment, losing only one or two patients in a hundred; but the statistics of the disease show that wherever it has been epidemic the average mortality has been nearly the same, in tropical and in temperate climates, on the old continent and in the new.

Dr. La Roche introduces the chapter on the pathology of yellow fever with the "painful avowal," that in regard to that important point, notwithstanding all the labor that has been bestowed upon it, little progress has been made by the profession. The following quotation expresses his theory of its pathology:—

"The cause of this complaint, however it may have originated, is a special poison, which, having penetrated the system and entered the blood vessels in a manner well known, is carried everywhere through the instrumentality of the blood. It produces in that fluid changes, to which attention was called in a former chapter, and others of a more intimate kind, which escape the cognizance of our senses; and by means of the polluted blood, occasions important morbid changes in some of the abdominal organs. It produces, besides, a morbid impression on the nervous centers; and these, as well as the former, in their turn, induce, by virtue of the numerous sympathies which bind together the various components of the system, diseased modifications in various organs and tissues, and thereby give rise to phenomena of a more or less peculiar kind, or essential to the manifestation of the fever, and varying somewhat in number and violence in different individuals and under different circumstances. The morbid actions thus produced may be combined with the opposite conditions of the vital forces, and may or may not be accompanied with inflammation of the parts primarily or secondarily affected; these and the other differences alluded to depending on age, habits of body, and numerous other circumstances connected with the individual; on the degree of concentration and violence of the efficient cause; on the peculiar character of the epidemic constitution of the atmosphere; on the nature of the localities, and numerous other modifying agencies. Hence, in some cases—under some circumstances, in the majority, if not in all—the morbific action of the poison is such as to destroy at once the powers of life; or, in less rapidly fatal instances, to greatly diminish them in all the parts to which it penetrates, and by that means, as well as by the diminution of fibrin or other changes occasioned in the blood, to impede or annul the force of reaction inherent in the system—impairing or destroying the vitality of the tissues and organs, checking the secretions, modifying and vitiating the power of nutrition, lowering the tonic force and vital energy of the blood-vessels, promoting the formation of capillary and venous congestions, facilitating the escape of blood from or in the substance of the tissues, etc."

"That the disease is a general one we may infer from a circumstance to which attention has sometimes been called by pathologists. Jaundice, as Mr. Maher remarks, constitutes a phenomenon of too prominent a kind to allow it to have been forgotten in the description of the disease. So far from this, it has been amply dwelt upon everywhere and by every writer, and has even given a name to the disease under consideration. Yet this symptom often fails to present itself. The same may be said of the black vomit, so that the yellow fever may really exist without being characterized by either of those symptoms. This proves to my mind, as Mr. Maher states, that the disease to which these two names have been given is not a local affection as these nomenclatures would lead us to infer. If the morbid changes were confined to the stomach and to the liver, the effects produced should always be the same, and the black vomit, as well as the jaundice, should never be absent. If, on the contrary, the lesion which constitutes the yellow fever is generalized and distributed at the same time to one or more apparatuses, we may readily understand that some of its phenomena of outward expression may not be fully developed without its being possible on that account to doubt the identity of the disease.* That the yellow fever does, like other great epidemics, give rise in the course of its progress to morbid changes in particular organs, no man of sense and common observation will deny; such effects are to be expected from all poisons which penetrate into the blood, or act in any way on the system. Sometimes in this disease there is gastritis; the liver is generally changed in a remarkable degree; the kidneys are found altered in color and texture; the lungs show dark patches; the brain is congested and bloody tumors form; the secretions are checked; the texture of the skin is so changed that I have seen the cuticle, by attempting to cleanse the face with a towel wiped cff and the skin left as raw as a blistered surface, and this, too, twelve hours before death. Who would say that small-pox and plague were localized because they affected the skin or glands, and so with the gastritis of yellow fever; nor is there any locality which can be assigned to inflammation which will account for the tendency to rapid collapse, the state of the blood, the depressing effects often seen from depletion, the early demand for stimulants."†

The second volume of this compendious treatise opens with an inquiry into the *cause* of yellow fever, which is pursued under the several heads of causes depending on the individual, hygienic causes, contagion, and infection. Under the first is considered *acclimatization*, the protective agency of which against the complaint is universally recognized. As to the time required to obtain the protection different opinions have prevailed, but attacks on citizens who have been for a number of years resident in places subject to yellow fever are, as Dr. La Roche expresses

* N. O. Jour., x. 467.

† Nott, Am. Jour., N. S., ix. 288.

it, "exceptions to a rule." The immunity, he believes, "is usually acquired in ten years—often in five—not unfrequently sooner, and is much more perfect when the individual has resided in the place during the prevalence of an epidemic, especially when the latter has been of a severe and malignant character, than when he has done so during a succession of healthy seasons."

Whether persons are likely to suffer a second time from yellow fever is still a disputed question. The general belief is that one attack shields the individual from all further danger; but there are physicians who contend that yellow fever is in no way different, in respect to its protective power, from the bilious remittent fever, which is well known not to exhaust the susceptibility of the system to the morbid impression of the efficient cause. The fact is, we suppose, that neither opinion is strictly correct, but that while a certain immunity is gained by one attack, subsequent attacks are of occasional occurrence, in other words, that the preservation, though the rule, is not universal and absolute.

In regard to age, temperament, sex, and race, as modifying causes of yellow fever, our author has collected many instructive facts. Persons of the sanguineous temperament, in vigorous health, seem to be more subject to the disease than individuals of a feeble constitution. Women are less obnoxious to the poison than men, and when they are seized by the disease it is in a milder form. The aged and the young are not as great sufferers as persons in middle life, which, as in the case of females, is probably due to their being less exposed to the infection. Negroes, especially those born and reared in countries where yellow fever is endemic, enjoy a comparative exemption from the malady, and, as has been remarked of the female sex, if seized with it at all, it is generally in a less malignant form. But those people newly arrived from Africa have at times suffered severely from the fever, and their acclimatization, like that of the whites, may be lost by long residence in cold climates.

Passing over all that relates to the other causes concerned in the generation of yellow fever, about which our author has written with his usual fullness and clearness, we come to consider what he terms "the efficient and immediate cause." In reference to this cause we need hardly say, the greatest diversity of opinion prevails. Some, nay very many professional observers, and the larger portion of the public, regard the disease as the product of contagion imported from abroad. By others the cause is held to be a febrile poison generated on the spot; while others again, though holding to its domestic origin, contend that it is developed by certain conditions of the atmosphere independent of malaria. The arguments in favor of each one of these opinions are given by Dr. La Roche at great length, but we shall not attempt to follow him in their presentation.

Much controversy has prevailed in our country, in years past, respecting the authorship of the non-contagion theory. Dr. Potter, Dr. Davidge, and Dr. Caldwell have all contended for that honor. Dr. La Roche proves conclusively that Dr. Deveze was the first physician in America to proclaim the non-contagious character and domestic origin of yellow fever. This he did in 1794, a year before Dr. Potter claimed to have broached the opinion to Dr. Rush, and three years before the publication of Dr. Davidge's first essay on the subject. About the same time Dr. Caldwell, along with Rush, Coxe, Physick, and Dewees, embraced the doctrine of domestic orgin, and a year afterwards expressed the belief that "the disease is not contagious in the West Indies, and rarely, if ever, so in the United States." But the doctrine, it seems, may be traced to remote times. Thus in 1643, Ligion, who had witnessed the epidemic at Barbadoes, expressed the belief that it had a domestic origin, and we are told by Lefort that the opinion of the non-contagion of yellow fever is not only general, but traditional in the West Indies. This is the opinion for which our author strenuously contends. From his long and very elaborate argument we can afford room to make but a single extract:—

"*Yellow Fever Local in its Habitation.*—Unlike contagious diseases generally, and similar to endemic fevers, though in a more marked degree, the yellow fever is local in its habitation. The first, when introduced into a place, extend in all directions, and with more or less rapidity diffuse themselves in any part of such places where the contagion may be carried, even at considerable distances from the original cases, each case which occurs there acting as a new source of contamination, and serving to widen the sphere of the epidemic. Endemic autumnal fevers, especially when assuming the epidemic form, while they retain their so universally recognized non-contagious character, pervade a wide extent of country—differing, however, from the former in this, that though spreading thus widely, they nevertheless do so within certain bounds and in certain directions only, and sometimes spare, more or less completely, localities in even close proximity to others severely infected.

"Now, if we examine what takes place in reference to the yellow fever, we discover that the sphere of its prevalence is always somewhat and on some occasions very circumscribed; the disease remaining confined within the limits of the localities where it originated, or extending at only a comparatively small distance from these. In Philadelphia the disease has, during its various visitations, exhibited in a most striking manner this inability to diffuse itself afar. In 1699 the fever which commenced at a wharf between Market street and the drawbridge, extended over the infant city, but does not appear to have gone much, if at all, beyond its precincts.* In 1747 it was mostly confined within the limits of the southern parts of the city below the drawbridge, and in the neighborhood of the dock, which was then uncovered.† As we have already seen, the fever in 1762 was mostly circumscribed between Pine street, northerly, and three or four squares southerly, and extended from Front or Water street to Third or Fourth in a westward direction. In the memorable epidemic of 1793 the disease, after commencing in Water street, between Mulberry and Sassafras, extended northwardly up Water to Vine, and the streets running east and west, and several running in a contrary direction; and finally in most parts of the city, which was then of limited extent, as also in the suburb of Southwark, in Kensington, at that time a separate village. Beyond these localities it did not spread.

"The next year its limits were more circumscribed, and were embraced within Market and Walnut in one direction, and Water and a few streets to the westward in the other. In 1797 the infection was chiefly confined to the district of Southwark, and to the village of Kensington. Some cases, it is true, appeared in the city proper; but most of these were easily traced to the above sources. The disease in 1798 spread much more extensively than it had done in preceding sickly periods—even perhaps than in 1793—every part of the city, as well as Southwark and Kensington, becoming, before a long time had elapsed, to a greater or less extent the seat of the calamity. But, as in other seasons, the disease remained circumscribed within the above limits, to which all cases that occurred beyond could be traced. In 1799 the epidemic spread from Penn street on the wharf, to almost every part of the

* See ante, vol. i. p. 53, Additional Facts, by Col. of Phys., p. 5.
† See ante, vol. i. p. 60, Additional Facts, by Col. of Phys., p. 6.

city east of Seventh street, beyond which very few cases occurred; and between Pine and Lombard streets, near the southern boundaries of the city, and the district of Southwark, near the Swedes' Church. In 1802 it extended from the corner of Vine and Front to other parts of the city, particularly in Front and Water streets, near the drawbridge. The next year it prevailed in Chestnut near Water street, in Water and Sassafras, and in the lower part of the city near South; but, with few exceptions, its range did not extend to the westward of Second street, and in many parts not even that far. In 1803 it prevailed in Water street, scarcely reaching Second in a form deserving the name of yellow fever. In Water street it occurred at various points embraced within the space of a mile.* Breaking out, in 1805, in the district of Southwark, between the New Market and the Swedes' Church, the disease extended in various directions. But the principal focus of the infection was circumscribed within the limits of Southwark, and a small section of the southern extremity of the city proper east of Second street. In Southwark the disease extended to the east side of Fourth street. In 1819 it was strictly limited to two points—the north side of Market street wharf, and Front street above Walnut; no other cases occurring in any part of the city. In 1820 the fever, though appearing in several separate localities parallel with the river Delaware, but more or less distant from it—Water street near Sassafras, on the wharf near Walnut, in Water street between Market and Mulberry, in Front between Walnut and Chestnut, in Letitia Court, in Second street near Shippen, and finally in the Northern Liberties—did not reach to the east side of Second street. In 1853 it broke out at the corner of South street and the wharf, and continued during the season to prevail in that neighborhood, occupying an area of two or three squares in a northern and southern direction, and a third of that distance westwardly. The fever also broke out, but prevailed to a very limited extent, in the Northern Liberties, leaving the intermediate space, and all north of Spruce, free and healthy.

"That cases occurred beyond the limits ascribed to the disease, during the several epidemics mentioned, is certainly true; but in the very large majority of these, if not in all, they were in persons who had imbibed the seeds of the infection in the sickly districts.

"Nor is it in Philadelphia alone that the yellow fever is found to be thus circumscribed within a certain area. Turn we to New York, we find that in 1791 the disease was limited to Peck Slip and the neighboring streets—principally Water street—whence it spread over some portions of the then small city.† In 1795 it did not advance beyond the wharves and vicinity.‡ In 1798 New

* Caldwell, Med Repos., v i 149 † Addoms, Dissertat. Inaug., p. 7.

‡ Bayley, pp. 60–90; Seaman, in Webster's Col., p. 5; E. H. Smith, ib. p. 67.

Slip was the locality most affected.* Two years after it was limited to Pearl and Water streets, and the adjoining slips.† In 1803 the first alarm arose about the Coffee House Slip, and in that neighborhood. The fever prevailed about the same time in some other parts of the city, but principally near the margins of the two rivers.‡ In 1805 it was at first limited to the eastern side of the city, in Front, Water, and Pearl streets—principally below Burling Slip. After a short time, however, it broke out near the North River, and prevailed mostly on the low ground situated on the margin of the two streams.§ But the area of the infected district did not exceed two hundred yards from one extremity to the other. The fever of 1819 was confined within narrow limits, the infected district being described by the Board of Health as beginning at the foot of pier No. 8, East River; thence running on the eastern side of the same pier, and the adjoining slip to the corner of Pearl street; thence up Pearl to the west side of Wall street; thence down Wall street to the East River—thus including little more than the old slip and the parts adjacent."‖

After the discussion of contagion, which takes up nearly four hundred pages of his book, Dr. La Roche devotes a chapter to an inquiry into the nature of the yellow fever poison, but without very satisfactory results. The nature of the poison is still shrowded in profound mystery. We wish we could speak otherwise in regard to the treatment of yellow fever. We might be reconciled to our ignorance respecting its cause—whether a miasm, an infection, or a contagion—if, by our long experience, we had acquired satisfactory skill in its treatment; but when, after passing over all these ample pages so full of information on many points, we arrive at the concluding chapters, it is with a melancholy feeling that we review the various conflicting plans which have been tried, applauded, and then given up for others as unsatisfactory and unavailing. Nevertheless, progress has been made in this direction. Both in regard to treatment and prophylaxis we have gained much valuable knowledge, as the attentive reader of this work cannot fail to be convinced. The author, as a faithful his-

* McKnight, Med. Reg., iii. 295; Hardie, Fev. of 1798, p. 28; Rept. on Quarntine Laws, p. 8.

† Seaman, Med. Repos., iv. 250.

‡ Miller (E.), Works; Hardin on Fev. of 1822, p. 13; ib., Doc. relative to the Board of Health, p. 36.

§ Miller, Report in Document relative to Board of Healtn, p. 46, and Works, p. 89; Rodgers, Letter to Board of Health, in same doc., p. 25.

‖ Watts, pp. 302, 350; Med. Rep., N. S., v. 240.

torian, has given an account of all the numerous methods that have been proposed. One writer quoted speaks thus of mercury in forms of the fever where the mild antiphlogistic plan is ineffectual:—

"'But when,' as he says, 'the force of fever becomes higher and more destructive, it fails so often as to produce the necessity of employing more efficient means. It leaves so much to be done by the powers of nature—for really it professes no more than the removal of obstacles to the curative process, which is performed or attempted by them—that they cannot be confided in exclusively. The powers of nature, in such cases, are too feeble to resist and subdue the disease with their assistance alone. A remedy is demanded which can cut short or mitigate it, by translating excitement from a part which is so vital to one which is less so. *Sthenic* medicines aggravate the evil; *asthenic* medicines cannot reduce it, notwithstanding any reduction of the fluids or excitement; and blisters are incapable of diverting it. Under these circumstances, mercury has been resorted to with the most happy results; and although it does not succeed universally, it has filled up a considerable space in the deficiency of remedies.'"

The reader of the histories of modern yellow fever epidemics need not be told how this practice is condemned by the physicians of the present day. But we shall not pursue this subject.

We conclude by a hearty recommendation of this work to our professional brethren. Dr. La Roche has executed a task of which his friends, his profession, and his country may be proud.

